# Burden, risk factors and maternal and offspring outcomes of gestational diabetes mellitus (GDM) in sub-Saharan Africa (SSA): a systematic review and meta-analysis

**DOI:** 10.1186/s12884-019-2593-z

**Published:** 2019-11-28

**Authors:** Barnabas Kahiira Natamba, Arthur Araali Namara, Moffat Joha Nyirenda

**Affiliations:** 1 MRC/UVRI and LSHTM Uganda Research Unit, Plot 51-59, Nakiwogo Road, PO Box 49, Entebbe, Uganda; 2 Department of Noncommunicable Diseases EpidemiologyFaculty of Epidemiology and Population Health, London School of Hygiene and Tropical Medicine, London, UK

**Keywords:** Gestational diabetes mellitus (GDM), Sub-Saharan Africa (SSA), Burden, Risk factors, Outcomes, Prevalence, Overweight and obesity

## Abstract

**Background:**

The burden, determinants and outcomes of gestational diabetes mellitus (GDM) in sub-Saharan Africa are not known. We summarized existing evidence on the prevalence, risk factors and complications of GDM in the region.

**Methods:**

PubMed was searched from inception to January 31st 2019. Studies were included if carried out in any of the sub-Saharan Africa countries and were available as abstracts or full texts. Interventional studies and those only including qualitative data were excluded. We employed random effects modelling to estimate the pooled GDM prevalence and risk ratios (RRs) for risk factors and outcomes of GDM and their 95%CI.

**Results:**

283 papers were identified in the initial search, 33 of which met the inclusion criteria. Data on GDM burden suggest a pooled prevalence of 9% (95%CI, 7–12%). Family history of type 2 diabetes and previous history of GDM, macrosomia, stillbirth and abortion were important risk factors of GDM. In addition, being overweight or obese, over 25 years of age or hypertensive increased the risk of GDM. In terms of complications, GDM more than doubles the risk macrosomia (RR; 95%CI: 2.2; 1.1–4.4).

**Conclusions:**

There is a high burden of gestational diabetes mellitus in sub-Saharan Africa, but more studies are needed to document locally important risk factors as well as maternal and offspring outcomes. Interventions to reduce obesity among older African women might lead to reduced risk of GDM in sub-Saharan Africa.

## Background

Gestational diabetes mellitus (GDM) is defined as “any degree of glucose intolerance that sets in or is first diagnosed during pregnancy” [1]. Estimates suggest that GDM prevalence is 7.0% in North America [2], 5.4% in Europe [3] and 11.5% in Asia [4]. Differences in GDM prevalence across regions are, at least in part, due to methodological variations as there is currently little consensus on the appropriate methods to screen and diagnose GDM [1, 5–12]. Two-step screening and diagnosis methods, for example, are based on measurement of glucose concentration following a 50 g glucose challenge test (GCT) and then again after a 100 g oral glucose tolerance test (OGTT) [13]. On the other hand, one-step approaches only rely on the OGTT. In 2010, the International Association of Diabetes in Pregnancy Study Groups (IADPSG) endorsed a more stringent one-step screening and diagnostic criteria using 75 g OGTT [9] and this recommendation was adopted by the WHO in 2013 [12]. Adoption of the IADPSG 2010/WHO 2013 criteria is growing, although use of the other criteria still exists in many contexts.

There are limited data on the burden of GDM in sub-Saharan Africa (SSA). In 2015, a review by Mwanri and colleagues suggested a prevalence of 14% among high risk individuals [14], but the prevalence in the general population is largely unknown. Similarly, the risk factors for GDM among Africans have not been adequately documented. Classical factors such as maternal age, overweight or obesity and family history for type 2 diabetes have been reported to be important risk factors of GDM in SSA [14], as they are in other populations [4]. It is possible that other local drivers such as malnutrition and infections may play a role, although these have not been sufficiently explored [15]. There is increasing evidence that undernutrition in early life can lead to later risk of cardio-metabolic disorders like diabetes [16]. Similarly, chronic infections (such as in HIV or TB that are highly prevalent in the region), perhaps via inflammation and immune activation, are thought to increase risk of diabetes [17].

GDM is known to adversely impact maternal and offspring outcomes [18]. Infants born to GDM women are more likely to be macrosomic i.e. birthweight ≥4.0kgs [19]. Macrosomic infants are more likely to suffer from birth-related injuries such as shoulder dystocia. They are also more likely to be admitted to the neonatal intensive care unit with metabolic complications [20]. Because of increased baby weight, women with GDM are more likely to deliver by caesarean section (CS) and to suffer from vaginal lacerations and postpartum haemorrhage. Most women with GDM revert to normal glycaemic status after giving birth, but they remain at increased risk of developing type 2 diabetes in the long term [2].

Since the review by Mwanri and colleagues was published, a number of studies have been published assessing the burden or risk factors of GDM in SSA (such as [21–24]); thus, there is need for integrating these new findings into what is already known from previous efforts. Furthermore, much as some studies have examined maternal and offspring outcomes of GDM in SSA, to our knowledge, no one has comprehensively summarised this evidence. Therefore, in this paper, we will provide a current update integrating new evidence on the burden and determinants of GDM in SSA (including the extent to which each identified risk factor increase GDM risk), as well as undertake a rigorous review of the impacts of GDM on maternal and offspring outcomes.

## Methods

### Search strategy and selection criteria

This systematic review and meta-analysis was registered with PROSPERO (2019: CRD42019116853) and carried following the Preferred Reporting Items for Systematic Reviews and Meta-Analyses (see filled PRISMA checklist in Additional files 1, [25]. We searched PubMed with the following search (MeSH) terms: (diabetes mellitus) AND (pregnancy) AND (africa south of the saharah). We used a mixture of expanded MeSH terms and free-text words which are highlighted in Additional file 2. Thereafter, reference lists of relevant original research and review articles were looked into for more articles that suit our inclusion criteria. Further, additional studies were found through reverse-forward citation tracking i.e. checking recent publications and their references.

We included in this review any studies that: 1) were conducted in SSA countries according to the United Nations Statistics Division [26]; 2) reported prevalence or risk factors or outcomes of GDM as primary results; 3) were peer reviewed articles published in journals from inception to January 31st 2019; and, 4) had a sample size ≥100 participants. We excluded from this review: 1) interventional studies including quasi-experimental studies and randomized trials; 2) case-series or case reports; 3) studies only including qualitative data, editorials, comments, letters and systematic reviews; and, 4) non-peer reviewed studies; or, 5) animal research.

Relevant articles were identified from the search and then brought into EndNote version X7 after which duplicates were removed. The first two authors (BKN & AAN) separately screened titles and abstracts to identify potentially eligible articles per the previously stated inclusion and exclusion criteria. Where there was no GDM prevalence (or risk factors or outcomes) information in the title or abstract, the reviewers examined the entire full text. Further deliberations were held with the senior author (MJN) to resolve any disagreements for a final consensus before including the full text article in the present review.

We employed the 22-item “Strengthening the Reporting of Observational Studies in Epidemiology (STROBE) checklist” [27] to assess the quality of included studies and guided by the published detailed explanation on how to use the checklist [28]. Two independent assessors (BKN & AAN) evaluated the quality of included studies. The assessors discussed their scores and where they did not agree involved the senior author (MJN) in the discussion to reach a consensus. A quality assessment score out of 22 was determined for each study by assigning a point per addressed STROBE item; lower scores indicate relatively poor quality studies when compared to articles with higher scores. Studies scoring 14 or greater on the STROBE checklist were retained for further analyses while those scoring less than 14 were dropped.

BKN recorded the data from studies of moderate to high quality meeting our inclusion criteria into a data extraction form using Excel®, while AAN confirmed the correctness and comprehensiveness of the extracted data. The following study features were extracted: first author, year of publication, country, screening and diagnostic criteria for GDM, sample size and number of GDM cases and STROBE score. Other collected data important to risk factor analyses were the number of GDM cases exposed to a given risk factor (as well as the total number of exposed subjects) and number of cases unexposed to the risk factor (as well as the total number of unexposed subjects). To examine the adverse impacts of GDM, we also noted down the number of cases of the outcome (e.g. macrosomia or caesarian section births) exposed to GDM during the index pregnancy (as well the total exposed to GDM) and number of cases of the outcome not exposed to GDM (and the total unexposed to GDM).

### Data analysis

We employed the random-effects meta-analysis as described by DerSimonian and Liard [29] to pool data on the burden (primarily prevalence), risk factors and outcomes of GDM in SSA. We report pooled point estimates of the prevalence and risk ratios (RRs) and their 95%CIs for risk factor and outcome from included studies. The I^2^ index was used to assess heterogeneity across studies, higher I^2^ indicate increasing discrepancy due to variations across studies [30]. Meta-analyses for any of our studied outcomes or across subgroups were performed whenever there were at least 3 or more studies to combine; outcomes or subgroups with only two or fewer studies were not analyzed. For subgroups, we examined differences in GDM prevalence over time (studies published before 2009 and those from 2010 to 2018) and those using different diagnostic criteria (IADPSG/WHO2013 versus other criteria). Statistical analyses were conducted in STATA version 15 (StataCorp, College Station TX). Since some selected studies had prevalence estimates at the 0% bound, we employed the metaprop command with Freeman-Tukey double arcsine transformation to pool prevalence across studies [31] whereas the metan command [32] was used to determine the RR and 95%CIs for risk factors and outcomes of GDM in SSA.

## Results

**Fig. 1:**
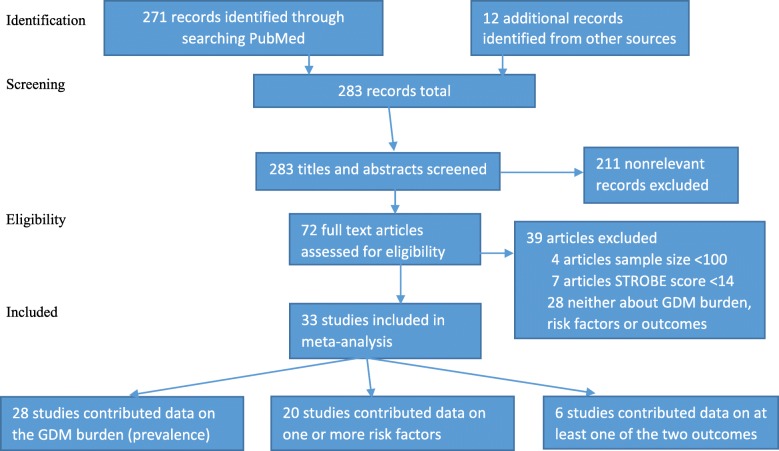
PRISMA flow diagram showing the search process

Initially, we identified 271 papers from PubMed (Fig. 1). Additional 12 papers were identified through reverse-forward (recent) citations, checking of reference lists of relevant original papers and other reviews papers, adding up to 283 papers. After applying our inclusion and exclusion criteria, we ended up with 33 eligible articles [21–24, 33–61] for inclusion in this systematic review. Of these, 28 papers contributed towards estimation of GDM prevalence [21, 23, 24, 33–38, 40–42, 46–55, 57–61], 20 towards assessment of risk factors of GDM [21–24, 39, 41, 44, 45, 47–50, 52–55, 57, 58, 60, 61] and 6 towards the evaluation of the impacts of GDM on maternal and offspring outcomes [39, 43, 47, 51, 57, 61].

The 33 papers in this review have a total sample size of 31,821 women and 2146 GDM cases from 12 SSA countries (Table 1). Further, the median (interquartile range) sample size of included studies is 368 (251–890) participants. Eleven studies were published between 1969 and 2009 and 22 studies from 2010 to 2018. In terms of quality of included studies, article scores on the STROBE checklist are summarized in Table 1. The median (interquartile range) STROBE score was 17 (15–18). The lowest STROBE score was 14 and the highest was 21 suggesting that included studies were of moderate to high quality.

**Table 1:** Characteristics of the 40 included studies and their respective STROBE scores

Author year [Ref]	Country	Screening procedure	Diagnostic criteria	50 GCT cut-off mmol/L	Fasting cut-off mmol/L	1 h cut-off mmol/L	2 h cut-off mmol/L	3 h cut-off mmol/L	1 or 2-steps	Sample size	GDM cases	STROBE Score
Notelovitz 1969 [33]	South Africa	Fasting, 2 h OGTT (100 g)	Own criteria, modified O’Sullivan & Mahan		6.4		8.3		One step	301	25	14
Jackson 1979 [34]	South Africa	Fasting, 1 h 2 h OGTT (50 g)	Own criteria, adapted O’Sullivan & Mahan		5.5	10.0	6.7		One step	558	17	14
Abudu 1987 [35]	Nigeria	50 g GCT, fasting, 1 h 2 h OGTT (50 g)	O’Sullivan & Mahan	7.2	5.0	9.2	8.1		Two steps	336	5	14
Swai 1991 [36]	Tanzania	Fasting, 2 h OGTT (75 g)	WHO 1985		7.8		11.1		One step	189	0	14
Ranchod 1991 [37]	South Africa	50 g GCT, fasting, 2 h OGTT (75 g)	WHO 1985	7.8			7.8		Two steps	1717	65	15
Seyoum 1999 [38]	Ethiopia	Fasting, 2 h OGTT (75 g)	WHO 1985				7.8		One step	890	33	17
Ozumba 2004	Nigeria	Fasting, 2 h OGTT (75 g)	WHO 2006		7		11.1		One step	5025	15	14
Olarinoye 2004 [40]	Nigeria	3 h OGTT (75 g)	WHO 1985				7.8		One step	138	16	17
Mamabolo 2007 [41]	South Africa	Fasting, 2 h OGTT (75 g)	WHO 2006		7.0		11.1		One step	262	4	18
Adegbola 2008 [42]	Nigeria	50 g GCT, 1 h, 2 h, 3 h OGTT (100 g)	CC criteria	7.2	5.3	10.0	8.6	7.8	Two steps	241	12	16
Kamanu 2009 [43]	Nigeria	50 g GCT, 1 h 2 h OGTT (75 g)	modified CC	7.8		10.0	8.6		Two steps	9040	140	18
Basu 2010 [44]	South Africa	Fasting or random blood	Institutional protocol		8.0				One step	767	14	18
Kuti 2011 [45]	Nigeria	Fasting 2 h OGTT (75 g)	WHO 1999		7.0		7.8		One step	765	106	18
Tandu-Mba 2012 [46]	DR Congo	Fasting only	ADA 2003/4		5.3				One step	108	8	14
Jao 2013 [47]	Cameroon	Fasting only	ADA 2010		5.3				One step	316	20	16
Anzaku 2013 [48]	Nigeria	50 g GCT, 2 h OGTT (75 g)	WHO 1985	7.8			7.8		Two steps	253	21	18
Mwanri 2013 [49]	Tanzania	Fasting, 2 h OGTT (75 g)	IADPSG		6.1		7.8		One step	910	119	20
Fawole 2013 [50]	Nigeria	Fasting, 2 h OGTT (75 g)	WHO 1999		7.0		7.8		One step	530	9	18
Minsart 2014 [51]	Djibouti	Fasting, 1 h 2 h OGTT (75 g)	IADPSG		5.1	10.0	8.5		One step	231	106	17
Olagbuji 2015 [52]	Nigeria	Fasting, 1 h 2 h OGTT (75 g)	IADPSG		5.1	10.0	8.5		One step	1059	91	19
Oppong 2015 [53]	Ghana	Fasting, 1 h 2 h OGTT (75 g)	WHO 2013		5.1	10.0	8.5		One step	399	37	19
Olagbuji 2017 [54]	Nigeria	Fasting, 1 h 2 h OGTT (75 g)	IADPSG		5.1	10.0	8.5		Two steps	280	44	17
Mapira 2017 [55]	Rwanda	Fasting only	ADA 2011		7.0				One step	288	24	18
Pastakia 2017 [56]	Kenya	Fasting, 1 h 2 h OGTT (50/75 g)	IADPSG		5.1	10.0	8.5		One step	616	18	16
Nakabuye 2017 [57]	Uganda	Fasting, 2 h OGTT (75 g)	WHO 2013		5.1		8.5		One step	251	76	17
Oriji 2017 [58]	Nigeria	Fasting, 1 h 2 h OGTT (75 g)	WHO 2013		5.1	10.0	8.5		One step	235	35	18
Adam 2018 [59]	South Africa	Fasting, 1 h 2 h OGTT, 75 g	WHO 2013		5.1	10.0	8.5		One step	529	141	15
Njete 2018 [21]	Tanzania	Fasting, 1 h 2 h OGTT, 75 g	WHO 2013		5.1	10.0	8.5		One step	333	65	20
Nhidza 2018 [60]	Zimbabwe	Fasting, 2 h OGTT, 75 g	WHO 2006		7.0		11.1		One step	150	10	14
Macaulay 2018a [24]	South Africa	Fasting, 1 h 2 h OGTT, 75 g	IADPSG		5.1	10.0	8.5		One step	1906	179	21
Macaulay 2018b [61]	South Africa	Fasting, 1 h 2 h OGTT, 75 g	WHO 2013		5.1	10.0	8.5		One step	741	83	21
Egbe 2018 [23]	Cameroon	Fasting, 1 h 2 h OGTT, 75 g	IADPSG		5.1	10.0	8.5		One step	200	41	18
Feleke 2017 [22]	Ethiopia	NR	NR	NR	NR	NR	NR	NR	NR	2257	567	17
								Total		**31,821**	**2146**	

*ADA* American Diabetes Association, *CC* Carpenter and Coustan, *GCT* Glucose challenge test, *IADPSG* International Association of Diabetes in Pregnancy Study Groups, *NR* Not reported, *OGTT* Oral glucose tolerance test, *WHO* World Health Organization, STROBE: Strengthening the Reporting of Observational Studies in Epidemiology

The IADPSG/WHO 2013 diagnostic criteria for GDM were the mostly used (in 13 studies); these were followed by the WHO 1985 to WHO 2006 criteria (10 studies) and then the O’Sullivan & Mahan criteria (or their adaptation by Carpenter and Coustan (CC) or the National Diabetes Data Group (NDDG)) in 5 studies. Fasting glucose (FG) concentrations alone were used to diagnose GDM in 4 studies. In one Ethiopian study [22], the screening and diagnostic criteria for GDM was not reported.

In terms of country of the study, 11 studies were from Nigeria alone [35, 39, 40, 42, 43, 45, 48, 50, 52, 54, 58], 8 studies from South Africa [24, 33, 34, 37, 41, 44, 59, 61] and 3 studies from Tanzania [21, 36, 49]. Cameroon [23, 47] and Ethiopia [22, 38] contributed two studies each. The other 7 countries (Democratic Republic of Congo [46], Djibouti [51], Ghana [53], Kenya [56], Rwanda [55], Uganda [57] and Zimbabwe [60]) contributed one study each.

### Prevalence of GDM in sub-Saharan Africa

**Fig. 2:**
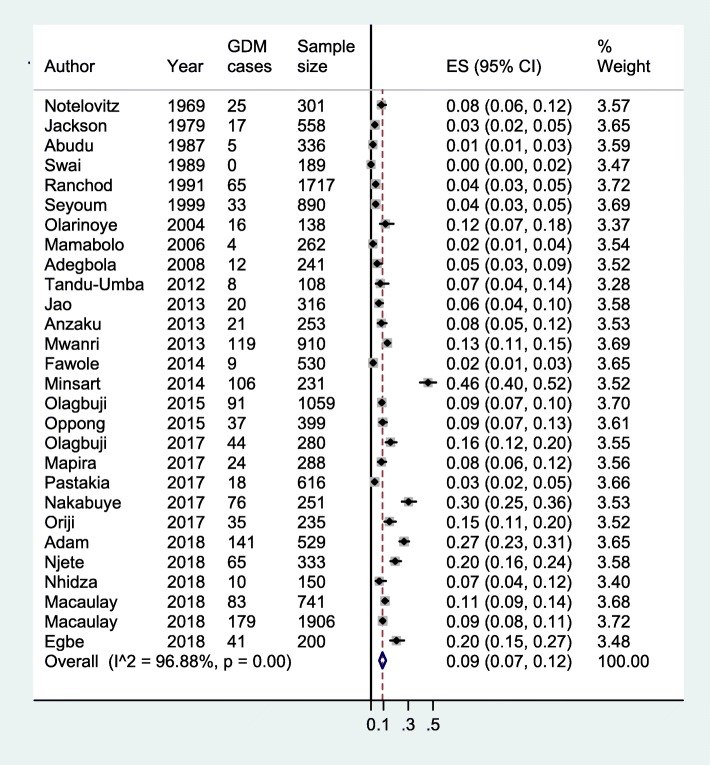
Prevalence of GDM in sub-Saharan Africa

Our meta-analysis combining data from 28 studies estimates the overall prevalence of GDM in SSA to be 9% (95%CI, 7–12%) (Fig. 2). Further subgroup analyses suggest that the GDM prevalence is 3% (2–5%) in studies published between 1969 and 2009 and 13% (9–17%) for studies from 2010 to 2018 (Additional file 3). Looking at the diagnostic criteria used in included studies, studies employing the O’Sullivan and Mahan method (or its modification by Carpenter and Coustan or the National Diabetes Data Group) suggest a GDM prevalence of 4% (2–75%); those using the WHO 1985 to WHO 2006 criteria have a combined prevalence of 4% (2–6%); and, those relying on fasting blood alone suggest a prevalence of 7% (6–9%). On the other hand, studies using the IADPSG or WHO 2013 criteria have a combined GDM prevalence of 16% (11–21%) (Additional file 4).

### Risk factors for GDM in sub-Saharan Africa

Twenty (20) included papers provide data on more than 14 different risk factors for GDM in SSA (with each risk factor having at least three different studies to combine), the results are summarized in Table 2, and details are given in Additional file 5. The most important risk factors for GDM in SSA based on the pooled analyses are history of GDM (5.9; 2.2–15.7), stillbirth (2.2; 1.4–3.4), macrosomia (1.8; 1.3–2.5) and abortion (1.8; 1.4–2.2) in prior pregnancies. Other important risk factors include family history of type 2 diabetes (1.8; 1.4–2.3) and hypertension (1.5; 1.2–2.1). Women older than 25 years (1.7; 1.2–2.4), those who are overweight or obese (1.6; 1.2–2.0) or multipara women (1.4; 1.1–1.8) were at increased risk of GDM. Being primigravida is significantly associated with a reduced risk of GDM (0.5; 0.3–0.9).

**Table 2:** Risk factors of GDM in sub-Saharan Africa

No.	Risk factor	No. studies included	RR	95%CI	I^2^	P heterogeneity
1	History of GDM	6	5.93	**2.24, 15.71**	92.90%	< 0.001
2	History of stillbirth	4	2.16	**1.36, 3.43**	36.80%	0.191
3	History of macrosomia	12	1.82	**1.31, 2.51**	63.40%	0.002
4	Family history of DM	16	1.79	**1.42, 2.25**	60.40%	0.001
5	History of abortion	3	1.78	**1.44, 2.19**	0.00%	0.852
6	Age > 25	11	1.70	**1.23, 2.36**	39.00%	0.089
7	BMI > 25	9	1.56	**1.20, 2.02**	61.10%	0.008
8	Hypertension	6	1.54	**1.16, 2.05**	0.00%	0.854
9	Multiparity	8	1.38	**1.05, 1.80**	66.40%	0.004
10	Primigravida	5	0.52	**0.29, 0.92**	72.80%	0.005
11	History of congenital anomaly	3	1.46	0.44, 4.83	0.00%	0.649
12	HIV status	4	1.14	0.90, 1.43	0.00%	0.990
13	Secondary or higher education	8	0.77	0.63, 1.02	38.90%	0.120
14	Physically active	3	0.36	0.07, 1.84	95.90%	< 0.001

*DM* Diabetes Mellitus, *GDM* Gestational Diabetes Mellitus, *BMI* Body mass index

Bold confidence intervals show significant risk factors

History of congenital anomaly in prior pregnancies (1.5; 0.4–4.8) and being HIV infected (1.1; 0.9–1.4) were associated with nonsignificant increases in the risk of GDM whereas having secondary or higher level of education (0.8; 0.6–1.1) or being physically active (0.4; 0.1–1.8) were associated with nonsignicant lower risks of GDM.

### Outcomes of GDM in sub-Saharan Africa

For only one maternal outcome (caesarian section (CS) delivery; 4 studies) and one offspring outcome (macrosomia; 5 studies) we found at least 3 or more studies to conduct a meta-analysis. We found that GDM results in a significant increase in the risk of giving birth to a macrosomic offspring (RR; 95%CI: 2.19; 1.08–4.43) as well as a nonsignificant increase in CS birth (1.14; 1.0–1.4) (Additional file 6). We did not find any SSA studies that examined the impact of GDM beyond the time when the offspring is born.

## Discussion

We estimate the prevalence of GDM in SSA to be 9% (95%CI: 7–12%) with risk factors that include having a family history of type 2 diabetes and previous pregnancies complicated by GDM, macrosomia, stillbirth and abortion. Factors such as being overweight or obese, or older than 25 years or hypertensive were associated with a higher risk of GDM. Lastly, GDM women have increased risk of macrosomia in comparison to those without GDM.

Our meta-analytic approaches suggested a combined GDM prevalence of 9%; however, there was a lot of heterogeneity (I^2^ = 96.9%, Fig. 2) among included studies. Possibly and because of this variability, differences in GDM prevalence can be seen in individual studies and exist across and within countries. It is as low as 0% in a Tanzania [36] and as high as 46% in Djibouti [51]. Even in the same country, different estimates of prevalence exist: 2 to 27% in South Africa [41, 59]. We aimed to investigate potential sources of variation in studies on GDM prevalence via sub-group analyses. Based on when the study was published (a proxy of when the study took place), it appears the prevalence of GDM has increased significantly since around 2010. This increase may reflect a true increase in the burden of GDM, for example, because of increasing prevalence of risk factors such as obesity. High rates of overweight and obesity among African women have been reported in some contexts in SSA [62]. Another source of heterogeneity might relate to recent changes in how GDM is screened and diagnosed. Indeed, as demonstrated in this review and others [4], adoption of the IADPSG criteria in 2010 has greatly influenced estimates of GDM burden, significantly increasing the number and proportion of individuals diagnosed with the condition.

Subgroup analyses related to when the study was conducted or diagnostic criteria did not help to eliminate the significant heterogeneity across studies in the subgroups (I^2^ remained greater than 40% in most sub-analyses). We performed meta-regression analyses (data not shown) to identify any further factors majorly influencing the estimate of GDM prevalence in the region. Meta-regression in this case considered both study (sample) size and study quality (STROBE score); however, neither variable was found to significantly influence the estimate of GDM prevalence. Meta-regression was considered not appropriate for analyses on the risk factors and complications associated with GDM. This is because there were very few studies included in each risk factor or complication analysis, and meta-regression requires many studies to implement [63]. Epidemiological approaches, rather than statistical methods, will be required to reduce variation across studies on GDM burden, determinants and complications in SSA. These will include, for example, more collaborative research, standardization of protocols and methodologies and studies conducted in more than one site within and across all SSA countries.

The estimated GDM prevalence in this review is lower than the 14% prevalence reported by Mwanri and colleagues for high risk women in SSA [14]; this should be expected since our analyses were not restricted to the risk profile of participants in included studies. The combined prevalence of GDM this review is also lower than that reported for Asia (11.5%) [4], but higher than that observed in European studies (5^.^4%) [2, 3]. These discrepancies could be due to methodological variations, but may also reflect differences in susceptibility to GDM in different populations. For instance, it has been suggested that Asian women are more likely to develop GDM than their Caucasian or African-American counterparts [64].

Most of the identified risk factors for GDM in SSA (such as family history of type 2 diabetes, obstetric history factors, age and BMI category) are well-known determinants of GDM risk and have been studied in other contexts [4]. The direction and magnitude of effects of these factors would have been expected a priori; and, these classical factors will continue to guide risk factor based approaches to screening for GDM in SSA. However, few existing studies have examined non-classical risk factors for GDM among SSA populations. For example, there continues to be limited data exist on the impact of exposure to in-utero and early childhood undernutrition or chronic infections (such as HIV, malaria and others) and lifestyle factors (such as local patterns of smoking, alcohol and dietary intake) on GDM risk in SSA.

We found that GDM is significantly associated with increased risk of macrosomia and a non-significant increase in the risk of CS delivery. This is in accord with well-established literature [19]. However, delivery of large babies may represent a particular problem SSA contexts, where the burden of cephalopelvic disproportion is already high and access to obstetric and early neonatal care are still a major challenge [65].

This is the largest systematic review (to present) on the burden and risk factors of GDM in SSA. It is also the first to systematically summarize the risk that GDM poses on maternal and offspring outcomes. Although we only searched PubMed because it is publicly available and accessible to us, our paper includes more moderate to high quality studies than previous efforts on the topic [14]. Even then, there are still very few studies of good quality conducted in SSA (for example in comparison to studies carried out in Asia [4]) and most of the available evidence was generated from Nigeria and South Africa. Also, there were not enough SSA studies to combine and assess the impact of GDM on most neonatal morbidities including macrosomia or CS births [66] or on maternal and offspring outcomes that happen well after the neonatal period (such as risk of type 2 diabetes [2], infant adiposity [67] or breastfeeding rates [68]). As scientific awareness and attention to GDM increases in Africa, new high quality studies documenting the burden, risk factors and complications of GDM and in a breadth of African countries will emerge. This will enable future systematic reviewers to be more selective and report less variability across retrieved studies when estimating the burden, risk factors and impacts of GDM in the region.

## Conclusions

Findings from this review suggest a GDM prevalence of 9% in SSA and that GDM is, to a large extent, driven by classical risk factors of the disease in other contexts. Although there are limited data on neonatal outcomes, macrosomia appears to be a common complication. More SSA studies are clearly required to rigorously document trends in GDM prevalence, characterize risk factors (both classical and emerging) and to better understand impacts on the mother and her offspring.

## Supplementary information

**Additional file 1.** PRISMA checklist.

**Additional file 2.** Search strategy.

**Additional file 3.** Prevalence of GDM in sub-Saharan Africa, overall and by year of publication.

**Additional file 4.** Prevalence of GDM in sub-Saharan Africa, overall and by diagnostic criteria.

**Additional file 5.** Risk factors of GDM in sub-Saharan Africa

**Additional file 6.** Maternal and offspring outcomes of GDM in sub-Saharan Africa.

## Data Availability

The datasets used and/or analyzed during the current study are available from the corresponding author on reasonable request.

## Abbreviations

CC: Carpenter and Coustan
CS: Caesarian Section birth
FG: Fasting Glucose
GCT: Glucose Challenge Test
GDM: Gestational Diabetes Mellitus
IADPSG: International Association of Diabetes in Pregnancy Study Groups
NDDG: National Diabetes Data Group
OGTT: Oral Glucose Tolerance Test
RR: Risk Ratio
SSA: Sub-Saharan Africa
STROBE: Strengthening the Reporting of Observational Studies in Epidemiology
WHO: World Health Organization
