# Supplementary material for: Burden, risk factors and maternal and offspring outcomes of gestational diabetes mellitus (GDM) in sub-Saharan Africa (SSA): a systematic review and meta-analysis
Source: BMC Pregnancy Childbirth. 2019 Nov 28;19:450. doi: 10.1186/s12884-019-2593-z (PMC6883645; doi:10.1186/s12884-019-2593-z)
Supplement: Supplementary file 2 — Additional file 2. Search strategy. [file 12884_2019_2593_MOESM2_ESM.docx]

**Additional file 2a. Search strategy in PubMed**

|  | **Area** | **Query** |
| --- | --- | --- |
| **#1** | **Abnormal glucose metabolism** | “Diabetes” or “glucose intolerance” or “hyperglycaemia” |
| **#2** | **Population** | “Pregnancy” or “pregnant” or “pregnan*” |
| **#3** | **Country** | “Africa, south of the saharah” or country specific terms (e.g. Nigeria, South Africa, Tanzania etc.) |
| **#4** | **Limits** | From inception to 31^st^ January 2019 |

**Additional file 2b. One example of PubMed search code**

("diabetes mellitus"[MeSH Terms] OR diabetes mellitus[Text Word] OR diabetes[Text Word]) AND ("pregnancy"[MeSH Terms] OR pregnancy[Text Word]) AND ("africa"[MeSH Terms] OR africa[Text Word] OR "africa south of the sahara"[MeSH Terms] OR africa south of the sahara[Text Word] OR "africa, western"[MeSH Terms] OR west africa[Text Word] OR "africa, eastern"[MeSH Terms] OR east africa[Text Word] OR "africa, southern"[MeSH Terms] OR southern africa[Text Word] OR "africa, central"[MeSH Terms] OR central africa[Text Word] OR "angola"[MeSH Terms] OR angola[Text Word] OR "benin"[MeSH Terms] OR benin[Text Word] OR "botswana"[MeSH Terms] OR botswana[Text Word] OR "burkina faso"[MeSH Terms] OR burkina faso[Text Word] OR "burundi"[MeSH Terms] OR burundi[Text Word] OR "cameroon"[MeSH Terms] OR cameroon[Text Word] OR "cabo verde"[MeSH Terms] OR cape verde[Text Word] AND "central african republic"[MeSH Terms] OR central african republic[Text Word] OR "chad"[MeSH Terms] OR chad[Text Word] OR "comoros"[MeSH Terms] OR comoros[Text Word] OR "congo"[MeSH Terms] OR congo[Text Word] OR "democratic republic of the congo"[MeSH Terms] OR Democratic Republic of the Congo[Text Word] OR "cote d'ivoire"[MeSH Terms] OR Cote d'Ivoire[Text Word] OR "djibouti"[MeSH Terms] OR djibouti[Text Word] OR "equatorial guinea"[MeSH Terms] OR equatorial guinea[Text Word] OR "eritrea"[MeSH Terms] OR eritrea[Text Word] OR "ethiopia"[MeSH Terms] OR ethiopia[Text Word] OR "gabon"[MeSH Terms] OR gabon[Text Word] OR "gambia"[MeSH Terms] OR the gambia[Text Word] OR "ghana"[MeSH Terms] OR ghana[Text Word] OR "guinea"[MeSH Terms] OR guinea[Text Word] OR "guinea-bissau"[MeSH Terms] OR Guinea-Bissau[Text Word] OR "kenya"[MeSH Terms] OR kenya[Text Word] OR "lesotho"[MeSH Terms] OR lesotho[Text Word] OR "liberia"[MeSH Terms] OR liberia[Text Word] OR "madagascar"[MeSH Terms] OR madagascar[Text Word] OR "malawi"[MeSH Terms] OR malawi[Text Word] OR "mali"[MeSH Terms] OR mali[Text Word] OR "mauritius"[MeSH Terms] OR mauritius[Text Word] OR "comoros"[MeSH Terms] OR mayotte[Text Word] OR "mozambique"[MeSH Terms] OR mozambique[Text Word] OR "namibia"[MeSH Terms] OR namibia[Text Word] OR "niger"[MeSH Terms] OR niger[Text Word] OR "nigeria"[MeSH Terms] OR nigeria[Text Word] OR "reunion"[MeSH Terms] OR reunion[Text Word] OR "rwanda"[MeSH Terms] OR rwanda[Text Word] OR "sao tome and principe"[MeSH Terms] OR sao tome and principe[Text Word] OR "senegal"[MeSH Terms] OR senegal[Text Word] OR "seychelles"[MeSH Terms] OR seychelles[Text Word] OR "sierra leone"[MeSH Terms] OR Sierra Leone[Text Word] OR "somalia"[MeSH Terms] OR somalia[Text Word] OR "south africa"[MeSH Terms] OR south africa[Text Word] OR "sudan"[MeSH Terms] OR sudan[Text Word] OR "south sudan"[MeSH Terms] OR south sudan[Text Word] OR "swaziland"[MeSH Terms] OR swaziland[Text Word] OR "togo"[MeSH Terms] OR togo[Text Word] OR "uganda"[MeSH Terms] OR uganda[Text Word] OR "tanzania"[MeSH Terms] OR tanzania[Text Word] OR zanzibar[Text Word] OR "zambia"[MeSH Terms] OR zambia[Text Word] OR "zimbabwe"[MeSH Terms] OR zimbabwe[Text Word]) AND "humans"[MeSH Terms]
