# Supplementary material for: Burden, risk factors and maternal and offspring outcomes of gestational diabetes mellitus (GDM) in sub-Saharan Africa (SSA): a systematic review and meta-analysis
Source: BMC Pregnancy Childbirth. 2019 Nov 28;19:450. doi: 10.1186/s12884-019-2593-z (PMC6883645; doi:10.1186/s12884-019-2593-z)
Supplement: Supplementary file 3 — Additional file 3. Prevalence of GDM in sub-Saharan Africa, overall and by year of publication. [file 12884_2019_2593_MOESM3_ESM.docx]

**Additional file 3. Prevalence of GDM in sub-Saharan Africa, overall and by year of publication**
