# Supplementary material for: Burden, risk factors and maternal and offspring outcomes of gestational diabetes mellitus (GDM) in sub-Saharan Africa (SSA): a systematic review and meta-analysis
Source: BMC Pregnancy Childbirth. 2019 Nov 28;19:450. doi: 10.1186/s12884-019-2593-z (PMC6883645; doi:10.1186/s12884-019-2593-z)
Supplement: Supplementary file 5 — Additional file 5. Risk factors of GDM in sub-Saharan Africa [file 12884_2019_2593_MOESM5_ESM.docx]

**Additional file 5. Risk factors for GDM in sub-Saharan Africa**

1. History of GDM in prior pregnancies

ne/Ne: number of exposed GDM cases over total exposed to history of GDM in prior pregnancies; nu/Nu: number of unexposed GDM cases over total unexposed to history of GDM in prior pregnancies.

1. History of stillbirth in prior pregnancies

ne/Ne: number of exposed GDM cases over total exposed to history of stillbirth in prior pregnancies; nu/Nu: number of unexposed GDM cases over total unexposed to history of stillbirth in prior pregnancies.

1. History of macrosomia in prior pregnancies

ne/Ne: number of exposed GDM cases over total exposed to history of macrosomia in prior pregnancies; nu/Nu: number of unexposed GDM cases over total unexposed to history of macrosomia in prior pregnancies

.

1. Family history of type II diabetes mellitus

ne/Ne: number of exposed GDM cases over total exposed to family history to type II diabetes mellitus; nu/Nu: number of unexposed GDM cases over total unexposed to family history to type II diabetes mellitus.

1. History of abortion in prior pregnancies

ne/Ne: number of exposed GDM cases over total exposed to history of abortion in prior pregnancies; nu/Nu: number of unexposed GDM cases over total unexposed to history of abortion in prior pregnancies.

1. Age greater than 25 years

ne/Ne: number of exposed GDM cases over total exposed to age greater than 25 years; nu/Nu: number of unexposed GDM cases over total unexposed to age greater than 25 years.

1. BMI greater than 25kg/meter squared

ne/Ne: number of exposed GDM cases over total exposed to being overweight or obese; nu/Nu: number of unexposed GDM cases over total unexposed to being overweight or obese.

1. Hypertension

ne/Ne: number of exposed GDM cases over total exposed to hypertension (chronic or during the index pregnancy); nu/Nu: number of unexposed GDM cases over total unexposed to hypertension (chronic or during the index pregnancy).

1. Multiparity

ne/Ne: number of exposed GDM cases over total exposed to being multipara; nu/Nu: number of unexposed GDM cases over total unexposed to being multipara

1. Primigravida

ne/Ne: number of exposed GDM cases over total exposed to being primigravida; nu/Nu: number of unexposed GDM cases over total unexposed to being primigravida.

1. History of congenital anomaly in prior pregnancies

ne/Ne: number of exposed GDM cases over total exposed to history of congenital anomaly in prior pregnancies; nu/Nu: number of unexposed GDM cases over total unexposed to history of congenital anomaly in prior pregnancies.

1. HIV exposed

ne/Ne: number of HIV-exposed GDM cases over total exposed to HIV; nu/Nu: number of HIV-unexposed GDM cases over total unexposed to HIV.

1. Having secondary or higher education level

ne/Ne: number of exposed GDM cases over total exposed to secondary or higher level of education; nu/Nu: number of unexposed GDM cases over total unexposed to secondary or higher level of education.

1. Physically active vs inactive

ne/Ne: number of physically active GDM cases over total who are physically active; nu/Nu: number of physically inactive GDM cases over total who are physically inactive.
