# Supplementary material for: Burden, risk factors and maternal and offspring outcomes of gestational diabetes mellitus (GDM) in sub-Saharan Africa (SSA): a systematic review and meta-analysis
Source: BMC Pregnancy Childbirth. 2019 Nov 28;19:450. doi: 10.1186/s12884-019-2593-z (PMC6883645; doi:10.1186/s12884-019-2593-z)
Supplement: Supplementary file 6 — Additional file 6. Maternal and offspring outcomes of GDM in sub-Saharan Africa. [file 12884_2019_2593_MOESM6_ESM.docx]

**Additional file 6. Maternal and offspring outcomes of GDM in sub-Saharan Africa**

1. Macrosomic offspring

ne/Ne: number of exposed macrosomic infants over total exposed to GDM; nu/Nu: number of unexposed macrosomic infants over total unexposed to GDM.

1. Caesarean section birth

ne/Ne: number of exposed CS births over total exposed to GDM; nu/Nu: number of unexposed CS births over total unexposed to GDM.
